# Tuberculosis case finding and isoniazid preventive therapy among people living with HIV at public health facilities of Addis Ababa, Ethiopia: *a cross-sectional facility based study*

**DOI:** 10.1186/1471-2458-14-52

**Published:** 2014-01-18

**Authors:** Amenu Wesen Denegetu, Bethabile Lovely Dolamo

**Affiliations:** 1 DLitt et Phil in Health Studies from University of South Africa, STOP Consultant, WHO/South Sudan, P.O. Box: 22751, Addis Ababa, Ethiopia; 2 Department of Health Studies, University of South Africa, P.O. Box 392 UNISA, 0003, Pretoria, South Africa

**Keywords:** HIV, INH preventive therapy, PLHIV, Screening, Tuberculosis

## Abstract

**Background:**

Activities to decrease the burden of tuberculosis (TB) among people living with HIV (PLHIV) include intensified TB case-finding (ICF), Isoniaizid (INH) preventive therapy (IPT) and infection control in health-care and congregate settings (IC). Information about the status of collaborative TB/HIV care services which decreases the burden of TB among PLHIV in Ethiopia is limited. The purpose of the study was to assess TB case finding and provision of IPT among PLHIV in Addis Ababa.

**Methods:**

A cross sectional, facility-based survey was conducted between June 2011 and August 2011. Data was collected by interviewing 849 PLHIV from ten health facilities in Addis Ababa. Both descriptive and inferential statistics were used to analyze findings and the results are described in this report.

**Results:**

The proportion of PLHIV who have been screened for TB during any one of their follow-up cares was 92.8%. Eighty eight (10.4%) of the study participants have been diagnosed for TB during their HIV follow-up cares. PLHIV who had never been diagnosed for TB before they knew their positive HIV status were nearly four times more likely to be diagnosed for TB during follow-up cares than those diagnosed before (AOR [95% CI]: 3.78 [1.69-8.43]). Nearly a third (28.7%) of all interviewed PLHIV self reported that they had been treated with IPT.

**Conclusions:**

It can be concluded that ICF for TB and IPT among PLHIV in Addis Ababa need boosting. Hence, it is recommended to put into practice the national and global guidelines to improve ICF and IPT among PLHIV in the city.

## Background

The dramatic spread of HIV in the past few decades, particularly in sub-Saharan Africa, has been accompanied by a major increase in the number of new cases of tuberculosis (TB) [1]. In 2010, TB killed an estimated 1.68 million people, including 0.38 million deaths among TB patients who were HIV positive [2]. 

The proportion of TB and HIV co-infection is highest in African countries. Overall, the African region accounted for 82% of TB cases among people living with HIV (PLHIV) [3]. According to the global fund to fight AIDS, TB and malaria (GFATM) information note, the interaction between TB and HIV presents additional challenges to TB control. It is crucial to improve and strengthen TB/HIV collaborative activities to reduce the burden of TB in PLHIV and reduce the burden of HIV among TB patients [1].

Collaborative TB/HIV management is essential to ensure that HIV positive TB patients are identified and treated appropriately, and to prevent TB in HIV positive people. Activities to decrease the burden of TB among HIV patients include intensified TB case finding (ICF), for those without active TB, INH preventive therapy (IPT) and infection control (IC) in health-care and congregate settings [4,5].

According to the World Health Organization (WHO) [5] document recommendation, ICF for TB means regularly screening all people with or at high risk for HIV infection, or in congregate settings (such as mines, prisons, military barracks) for the symptoms and signs of TB, followed by prompt diagnosis and treatment. The most efficient approach to detecting more cases and with shortened duration of infectivity involves intensified case-finding in settings where HIV-infected people are concentrated.

The same document [5] recommends that IPT for TB can be safely given to PLHIV without TB, reducing the risk of developing TB by 33-67% for up to 48 months. It is currently recommended for all PLHIV in areas with a prevalence of latent TB infection >30%, and for all PLHIV with documented latent TB infection or exposure to an infectious TB case, regardless of where they live.

Information about the status of collaborative TB/HIV care services which decreases the burden of TB among PLHIV in Ethiopia is limited. Hence, the purpose of this study is to assess ICF for TB and IPT among PLHIV at public health facilities of Addis Ababa, Ethiopia.

## Research objectives

### General objective

• To assess collaborative TB/HIV care services decreasing the burden of TB among PLHIV in Addis Ababa, Ethiopia.

### Specific objectives

• To determine the proportion of PLHIV attending HIV care clinics who were screened for TB.

• To determine the proportion of PLHIV attending HIV care clinics who were diagnosed for TB during their follow-up cares.

• To determine the proportion of PLHIV who have ever been treated with IPT during their follow-up cares.

## Methods

### Design

This is a cross sectional, facility-based descriptive study which was conducted at two referral hospitals and eight health centres in Addis Ababa City, Ethiopia. One health facility from each of the ten sub-cities of the city was selected randomly and two hospitals were included conveniently out of the five referral hospitals administered under Addis Ababa City Administration Health Bureau.

### Settings

The study setting includes *Zewditu* Memorial and *Menelik II* hospitals which are HIV therapy and care referral hospitals, and ten health centres, namely: *Lideta, Yeka, Kazanchis, Nifas-Silk Lafto No-1, Woreda 7, Kality, Bole* and *Gulele*. The health facilities were used to assess the implementation of ICF for TB and provision of IPT among PLHIV attending HIV care clinics.

### Study participants

The study participants include PLHIV who were registered at HIV clinics and on their follow-up cares in the health facilities. In this study, children under the age of 15 were not included.

### Sampling method

The sample size was determined using single population proportion formula. A 50% TB/HIV co-infection was estimated for Addis Ababa aiming for maximum sample size, with marginal error of 5% and 95% confidence interval. The calculated sample size was 384.

$$n=Z\frac{\alpha^{2}}{2}\times p\frac{\left(1-p\right)}{d^{2}}=\frac{{\left(1.96\right)}^{2}\times 0.5\left(0.5\right)}{0.0025}=384$$

To eliminate the design effect, the sample size was doubled and 10% of the total sample size was added as contingency. Hence, the total sample size used in this study become (384 × 2) + 10*%* = 845. Randomly selected PLHIV attending HIV care clinics at the selected health facilities in their follow-up cares were interviewed at exit.

In this study, the researcher used probability proportional to size (PPS) of cumulative HIV patient load in the facilities to determine the sub-samples for each of the sites. It was confirmed from HIV registration books that, the patient load in health centres on average was less than the load in the hospitals. In addition, the commutative load of patients among the different health centres was similar and that for hospitals was also similar. Therefore, the researcher decided to take an equal number of sub-samples from each of the eight health centres and also equal number from the two hospitals. But the sampled population from the health centres was less than the samples from the hospitals as reasoned above.

According to the health facilities HIV registration books, the study health centres and hospitals had on average 1120 and 1435 cumulative HIV patients at the time of the study; respectively. Hence, based on PPS method, the calculated sampled population for each of the health centres was 80 and that for the hospitals was 103.

### Data collection

The data collection was done from June 2011 to August 2011. The questionnaire was administered using experienced interviewers. The questionnaire was adapted from the WHO guideline, prepared for monitoring and evaluation of TB/HIV activities [6]. The questionnaire has acceptable levels of reliability and is valid for the Ethiopian situation. This is because, the questionnaire is adapted directly from the global TB/HIV monitoring Interview Tool [6] which is in-line with the global policy on collaborative TB/HIV activities [4] and national TB/HIV collaborative activity guideline [7]. In addition, the researchers believe that the questionnaire is reliable as data was collected by direct interview of HIV patients after their consent and reassurance of confidentiality. In addition, the interviews were conducted by sociologists who have no direct relation with the healthcare system and hence reduce professional bias.

Ten data collectors administered the questionnaires simultaneously during the data collection period. The principal investigator provided daily supportive supervision to all data collectors by checking the completeness and consistency of the filled questionnaires. Study participants were enrolled randomly for the interview on exit for consecutive days until the allotted sample size filled for that particular facility. One data collector was assigned for one health facility, and a maximum of 10 interviews were conducted per facility per person per day to maintain the quality of the data.

For this study, the questionnaire was pre-tested on 12 participants by each of the data collectors from two health centres. The pre-test data was collected from facilities not included for this study.

Ethical clearance was obtained from the University of South Africa and the Addis Ababa City Administration Health Bureau. A support letter from the Addis Ababa City Administration Health Bureau and permission was obtained from heads of each of the study health facilities. The interview was undertaken after ascertaining written consents from each study participant. The questionnaire did not include any specific identifier of the interviewee.

### Data analysis

The Statistical Package for Social Sciences (SPSS) version 15.0 was used for data capturing and analysis. Both descriptive and inferential statistics were used for presenting the findings. Logistic regression was used to identify factors associated with *TB case finding* across the various independent variables including; *sex, marital status, educational status, length of being diagnosed for TB* and *on HAART* and *previous history of TB disease.*

## Results

A total of 849 PLHIV, who were under HIV care follow-ups at public health facilities in Addis Ababa City Administration, participated in the study. Out of the total respondents 592(69.7%) were females.

The mean age (± SD) of the study population was 33.9 ± 9.9 years. Six hundred and fifty eight (77.4%) of the participants were in the age group 25–49, 415 (48.9%) were married and 198 (23.3%) were singles, 701 (82.6%) were followers of Christian religion. Around 38.2% had completed high school study, 9.40% had at least a certificate after completing high school. The remaining 29.1% and 23.2% accounted for primary completed and having no formal education at the time of the study; respectively.

Two hundred twenty five (26.5%), 168 (19.8%), 159 (18.7%) and 153 (18.0%) were unemployed, house wives, self employed and privately employed, by occupation; respectively. Nearly three-fourth (74.6%) of the study participants had become at least one year since they knew their HIV positive status. Seven hundred and five (83%) of these clients had already started Anti-Retroviral Therapy (ART) in the health facilities. Of those put under highly active antiretroviral therapy (HAART), 451 (63.9%) were on ART for more than one year.

### Screening for tuberculosis

The proportion of interviewed PLHIV who have been screened for TB during any one visit of their follow-up cares was 92.8% (788) (Table 1). The result revealed that, 3.7% (31) of participants have self reported that they had never been screened for TB. Besides, 3.5% (30) of interviewed PLHIV could not be sure whether they were screened for TB disease or not; this of course, can be associated with patients’ knowledge on their health services or poor communication of healthcare providers.

**Table 1:** Collaborative TB/HIV care service deliveries among people living with HIV in Addis Ababa

**Variable**	**Frequency**	**Percent**
**TB screening offered at HIV care clinic**		
Yes	788	92.8
No	31	3.7
I do not know	30	3.5
Total	849	100.0
**Ever been diagnosed for TB after knowing HIV status**		
Yes	88	10.4
No	761	89.6
Total	849	100.0
**Ever been treated with IPT**		
Yes	244	28.7
No	605	71.3
Total	849	100.0

n = 849.

### Prevalence of tuberculosis among people living with HIV

As TB is one of the first opportunistic infection for PLHIV, screening of all HIV positive patients for TB increases the uptake of active TB cases. In this study, 10.4% (88) of study participants have self reported that they were diagnosed for active TB disease during their HIV care follow-ups (see Table 1). In other words, 88 active TB patients were identified out of the total 849 PLHIV starting from their first contact to the healthcare provider for either of HIV testing, care or treatment services.

Results of multiple logistic regression with stepwise selection show that those who were on HAART for more than one year were nearly four times more likely to be diagnosed for TB than those who were on HAART for less than six months, and the result was statistically significant [AOR (95%CI): 3.53(1.17-10.65)]. Similarly, those who had never been diagnosed for TB before they knew their HIV status were nearly four times more likely to be diagnosed for TB during follow-up cares than those diagnosed before, the results were also statistically significant [AOR (95%CI): 3.78(1.69-8.43)] (Table 2).

**Table 2:** Tuberculosis case finding among people living with HIV in Addis Ababa, Ethiopia

**Variable**	**Ever been diagnosed for TB after HIV-positive**		**Unadjusted odds ratio (95% CI)**	**Adjusted odds ratio(95% CI)**
	**Yes (88)**	**No (761)**		
**How long have you been diagnosed for HIV? **				
Less than 6 months	7	117	1.00	1.00
6 -11 months	5	86	0.97(0.29-3.17)	0.83(0.24-2.89)
1-3 years	30	270	1.86(0.79-4.35)	0.82(0.26-2.53)
More than 3 years	46	288	2.67(1.17-6.08)	1.11(0.35-3.49)
**How long have you been on HAART? **				
Less than 6 months	7	142	1.00	1.00
6 months-1 year	9	96	1.90(0.69-5.28)	2.18(0.66-7.14)
More than 1 year	63	388	3.29(1.47-7.36)	3.53(1.17-10.65)
Not yet started	9	135	1.35(0.49-3.73)	1.29(0.45-3.72)
**Ever been diagnosed for TB before HIV-positive**				
Yes	7	160	1.00	1.00
No	79	597	3.03(1.37-6.68)	3.78(1.69-8.43)
Do not remember	2	4	11.43(1.78-73.30)	14.71(2.17-99.54)

n = 849.

On the other hand, univariate analysis revealed that, those who have become >3 years since they knew their HIV positive status were nearly 3 times more likely to be diagnosed for TB than those who became less than 6 months, [Unadjusted OR (95%CI): 2.67(1.17-6.08)] (Table 2).

### Isoniazid preventive therapy

Nearly a third 28.7% (244) of all interviewed PLHIV self reported that they had been treated with IPT (Table 1).

Logistic regression analysis of INH preventive therapy among the various independent variables did not show any association.

## Discussion

HIV infection is the most powerful known risk factor for progression of Mycobacterium TB infection to active disease; increasing the risk of latent TB reactivation by 20-fold [8]. Therefore, simple questionnaires to screen for TB can be administered when people seek HIV services for the first time (e.g., voluntary counselling and testing, HIV care, etc.) and/or by community based organizations supporting people with HIV. ICF serves as the most important gatekeeper for the two other I’s (infection control and Isoniazid preventive therapy), facilitating rapid identification of TB suspects (allowing for triage and other steps to reduce TB transmission), and acting as the necessary first step for healthcare providers to confidently prescribe IPT to PLHIV who do not have active TB [5].

According to the global TB report of 2011, in 2010, 2.3 million PLHIV were screened for TB (up from 1.7 million in 2009) and 178,000 of those without active TB were enrolled for IPT (double the level achieved in 2009) [3]. The number of PLHIV who were screened for TB was equivalent to more than half (58%) of the reported number of people who were enrolled in HIV care worldwide in 2010. The number started on IPT was 12% (178 144/1 464 579) of the reported number of PLHIV newly enrolled in HIV care in 2010 [3].

In this study 92.8% of PLHIV have self reported that they had been screened for common signs and symptoms for TB by their healthcare providers (more than 2 weeks of cough, night sweats, weight loss and weakness). This result is slightly higher than that of an earlier study in the same place by Wesen and Mitike that shows 89.7% [9]. This might indicate that TB/HIV care service in Addis Ababa has improved over time. Besides, this result is higher than findings from previous studies in other African countries. For example, a study on integration of TB and HIV services in Sub-Saharan Africa by Andrea and Wafaa in 2010 shows that, 64% of newly enrolled persons with HIV infection or AIDS in the first quarter of 2009 were screened for TB. An average of 22% of these patients screened positive for TB, of whom an average of 12% received a diagnosis of TB and initiated treatment [10].

In addition, persons with HIV infection or AIDS remain at risk of TB throughout the course of their HIV disease, including after ART initiation [11,12]. Thus, screening for TB should be done routinely during each follow-up cares. Performance of TB screening at every clinical encounter can be challenging in resource-constrained environments, particularly at sites that serve large numbers of persons with HIV infection or AIDS and where continuity care is a new paradigm [10].

The steady improvement in proportion of PLHIV screened for TB in Addis Ababa (from 89.7% in 2008 [8] to 92.8% in the present study) is also consistent with findings from a study in South Africa. A study at 9 HIV care and treatment centres in Nelson Mandela Bay District, Eastern Cape shows that the proportion of newly enrolled persons with HIV infection or AIDS who were screened for TB increased from 73% in the first half of 2007 to 95% in the latter half of 2008, after the introduction of a clinical record that incorporated TB screening questions [13].

Besides, this finding is similar to an earlier national report. According to the progress report of the Federal Democratic Republic of Ethiopia towards the implementation of the UN Declaration of Commitment on HIV/AIDS, the proportion of HIV-positive patients who were screened for TB increased from 25% in 2007 to 55% in 2009 for the country [14].

Escalating TB case rates over the past few decades in many countries in sub-Saharan Africa and in parts of South East Asia are largely attributable to the HIV epidemic. Since mid-1980s, in many African countries, including those with well-organized programs, annual TB case notification rates increased by four fold, reaching peaks of >400 cases/100,000 populations [4].

The result in this study shows that 10.4% of PLHIV developed active TB during HIV follow-up cares. But previous finding from a similar study in the same place (Addis Ababa) in 2008 [9] was slightly higher (15.6%) than the current prevalence. Hence, this decrement through time might be attributed to the improvements in HIV treatment and care services in Addis Ababa.

However, the finding in this study for the prevalence of TB among PLHIV is very low compared to findings elsewhere. For example, a study in Hong Kong in 2005 reveals a prevalence of 39% [15]. In another study in Karachi, Pakistan, in 2007, the prevalence of TB among HIV patients is 30.2%, though the setting is different [16]. A study in Georgia in 2008 also shows a higher prevalence of TB among PLHIV; up to 22% of HIV positive individuals have active TB [17].

On the other hand, this study revealed that the probability of being diagnosed for TB is increasing the longer patients are on HAART; *i.e*., those who became more than one year on HAART were nearly four times more likely to be diagnosed for TB than those of less than six months. This finding is in contrary to the fact that ART is one of the most potent tools for preventing HIV-associated TB, as evidenced in previous studies [18,19]. Hence, this may not be fully explained in this study. But, the finding can be consistent with the low coverage of IPT (28.7%); whereby, majority of PLHIV have no access to TB prophylactic therapy. However, an earlier study reveals that, initial reduction for the risk of TB is not apparent [20].

Isoniazid Preventive Therapy for TB can safely be given to PLHIV without active TB disease, reducing the risk of developing TB by 33-67% for up to 48 months. It is currently recommended for all PLHIV in areas with a prevalence of latent TB infection >30%, or for all PLHIV with documented latent TB infection or exposure to an infectious TB case, regardless of where they live [5].

In addition, the efficacy of IPT has been well documented in the prevention of at least the first episodes of TB among PLHIV in different studies. For example, a meta-analysis by Woldehanna and Volmink [21] shows that the provision of IPT to persons with HIV infection even in the absence of ART reduced the incidence of TB by 33% overall and by 64% among individuals with positive tuberculin skin test (TST) results, compared with placebo. Besides, observational studies suggest that IPT reduces the risks of TB and death during early ART [22] and that IPT and ART in combination result in a greater reduction in TB risk than does either treatment alone [22,23].

Despite these evidences, implementation of IPT in HIV care and treatment programs in resource-constrained countries has been documented to be unacceptably low [24].

The finding from this study is not very far from this fact. The study revealed that less than a third (28.7%) of all PLHIV interviewed had been enrolled for IPT during their follow-up cares. However, the finding of this study is higher than previous findings from other countries and even in Ethiopia. For example, according to the 2011 global TB report [3], sampled data for Bangladesh revealed IPT coverage of only 5.4%, for Ethiopia 15.1%, for Myanmar 8.0%, and only 3% for Nigeria.

In general, provision of IPT among PLHIV is linked to operational challenges and Ethiopia is not exceptional. There is no national protocol of doing tuberculin skin test (TST) for PLHIV before enrolled to IPT. However, the use of TST could reduce the number of patients receiving IPT and the numbers needed to treat to prevent one case of active TB [25]. The other reason for the low coverage IPT in the current study could be fear of drug resistance by some clinicians, as evidenced in a previous report [26], which is in contrary to the WHO recommendation of IPT provision, reaffirming concerns regarding the development of drug resistance should not be a barrier to providing IPT [25].

This study did not include exploratory qualitative data and might introduce recall bias, as findings are dependent on patients’ self report, which can be considered as a limitation. However, the use of a maximum sample population and doubling the design effect coupled with the use of social science professionals for data collection to reduce professional bias can be considered as the strengths of the study.

## Conclusion

It can be concluded that screening for TB among PLHIV in Addis Ababa is being done optimally. Prevalence of TB among PLHIV seems low compared to previous findings both in Ethiopia and in other countries; which could not be explained in this study. In addition, provision of IPT is still low, though better than previous studies. Hence, ICF for TB and IPT among PLHIV in Addis Ababa, Ethiopia need boosting by adopting the available national and international guidelines. In addition, continuous support of healthcare providers through on-going trainings and experience sharing forums can be used as a tool for improving the implementation of ICF and IPT.

## Competing interests

Both authors declare that they have no competing interests.

## Authors’ contributions

AD designed the study, coordinated, supervised and analyzed the data. BD assisted in the design of the study and conducted analysis of the data. Both contributed in the write up of the document. Both authors read and approved the final manuscript.

## Pre-publication history

The pre-publication history for this paper can be accessed here:

http://www.biomedcentral.com/1471-2458/14/52/prepub
